# The scaled-invariant Planckian metal and quantum criticality in Ce_1−*x*_Nd_*x*_CoIn_5_

**DOI:** 10.1038/s41467-023-36194-9

**Published:** 2023-02-03

**Authors:** Yung-Yeh Chang, Hechang Lei, C. Petrovic, Chung-Hou Chung

**Affiliations:** 1 Physics Division, National Center for Theoretical Sciences, Taipei, 10617 Taiwan Republic of China; 2 Department of Electrophysics, National Yang-Ming Chiao-Tung University, Hsinchu, 300 Taiwan Republic of China; 3 Condensed Matter Physics and Materials Science Department, Brookhaven National Laboratory, Upton, NY 11973-5000 USA; 4 Present Address: Department of Physics and Beijing Key Laboratory of Opto-electronic Functional Materials & Micro-nano Devices, Renmin University of China, Beijing, 100872 People’s Republic of China

**Keywords:** Phase transitions and critical phenomena, Superconducting properties and materials

## Abstract

The mysterious Planckian metal state, showing perfect *T*-linear resistivity associated with universal scattering rate, 1/*τ* = *αk*_*B*_*T*/ℏ with *α* ~ 1, has been observed in the normal state of various strongly correlated superconductors close to a quantum critical point. However, its microscopic origin and link to quantum criticality remains an outstanding open problem. Here, we observe quantum-critical *T*/*B*-scaling of the Planckian metal state in resistivity and heat capacity of heavy-electron superconductor Ce_1−*x*_Nd_*x*_CoIn_5_ in magnetic fields near the edge of antiferromagnetism at the critical doping *x*_*c*_ ~ 0.03. We present clear experimental evidences of Kondo hybridization being quantum critical at *x*_*c*_. We provide a generic microscopic mechanism to qualitatively account for this quantum critical Planckian state within the quasi-two dimensional Kondo-Heisenberg lattice model near Kondo breakdown transition. We find *α* is a non-universal constant and depends inversely on the square of Kondo hybridization strength.

## Abstract (web summary)

The Planckian metal is a special case of a strange metal, in which the linear-in-temperature scattering rate reaches a universal limit. Here the authors study this state in a heavy-fermion superconductor in magnetic field and propose a microscopic mechanical based on quantum criticality of the Kondo hybridization.

## Introduction

Metallic behavior that goes against the Landau’s Fermi liquid paradigm for ordinary metals has commonly been observed in a wide variety of strongly interacting quantum materials and yet the emergence of such metals is poorly understood. This unconventional metallic or non-Fermi liquid (NFL) behavior often exists near a magnetic quantum phase transition, and shows “strange metal (SM)” phenomena with (quasi-)linear-in-temperature resistivity and singular logarithmic-in-temperature specific heat coefficient^1,2^.

The Planckian metal state constitutes a particularly intriguing class of SM states and has been observed in the normal state of unconventional superconductors, including cuprate superconductors^3^, iron pnictides and chalcogenides^4–8^, organic^9,10^ and heavy-fermion compounds^10–13^, and twisted bilayer graphene^14^. The heart of this puzzling state is that the universal *T*-linear scattering rate reaches the Planckian dissipation limit allowed by quantum mechanics, 1/*τ* = *αk*_*B*_*T*/ℏ with *α* ~ 1^10,15–17^. This intriguing observation leads to fundamental questions: Is *α* a universal constant independent of microscopic details^18^? It was estimated that the Planckian bound can be reached in the low-temperature strong-coupling limit of electron-phonon and marginal Fermi liquid systems^17^. In quantum critical systems, however, it is not clear if and how *α* depends on microscopic coupling constant at quantum critical point (QCP) and its link to the quantum critical scaling in observables. It was argued that in quantum critical systems with *T*-linear scattering rate, the Planckian time *τ*_*Pl*_ ≡ ℏ/*k*_*B*_*T* is reached naturally (*α* ~ 1) as a result of the scaled-invariant observables *F*(ℏ*ω*/*k*_*B*_*T*) = *F*(*ωτ*_*Pl*_) at criticality^17^. However, such expectation has not yet been confirmed by a microscopic mechanism in any quantum critical systems.

While it is challenging to address these questions in cuprates, pnictide and organic superconductors, significant experimental and theoretical progress have been made in heavy-fermion quantum critical superconductors^19^. A prototypical example of such systems is the Ce*M*In_5_ family with *M* = Co, Rh, Ir under field and pressure where superconductivity and non-Fermi liquid (NFL) normal state properties emerge due to competition between antiferromagnetism and Kondo correlation near a antiferromagnetic Kondo-breakdown (AF-KB) QCP^20–25^. In particular, the *T*-linear resistivity in CeCoIn_5_ was reported to exhibit Planckian dissipation (*α* ~ 1)^10^, and therefore the Co-115 family is well suited for this study. In this work, we focus on Nd-doped CeCoIn5^26^.

**Fig. 1:**
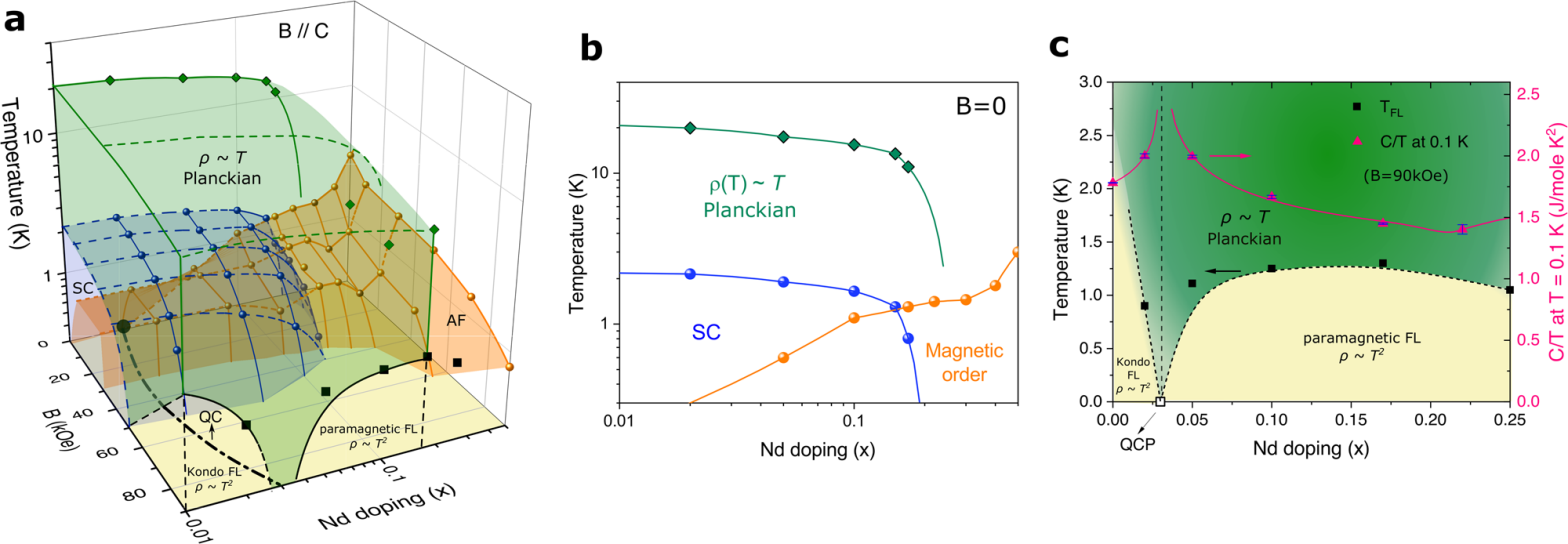
The phase diagram for Ce_1−*x*_Nd_*x*_CoIn_5_ for *B*∥*c*. **a** The (*x*,  *B*,  *T*) phase diagram: the antiferromagnetic (AF) Néel state occurs below the orange area and the superconducting (SC) phase sets in under the blue area (symbols). The data of zero-field plane is reproduced from Fig. 6 of ref. ^26^. A QCP is predicted on the doping axis near *x* ≈ 0.03 at zero field plane, hidden beneath the coexisting phase of the AF and SC phases. At temperature above the SC and AF phases and below the green region, this material shows linear-in-*T* strange-metallic (SM) behavior. A quantum critical (QC) line (black dotted-dashed line) is expected to exist at finite field and Nd doping, connected to the QCP at zero field, *x* = *x*_*c*_. **b** Phase diagram of **a** on the zero-field facet. **c** Phase diagram of the system at *B* = 90 kOe. As the external field is large enough (approximately *B* ~ 70 kOe for *B*∥*c*), a QCP emerges at *x* ≈ 0.03 as inferred from the *T*^2^ resistivity measurement (below *T*_*FL*_). This QCP is located at *B* = 90 kOe along the QC line of **a**. The enhancement of the normal-state electronic specific heat *C*/*T* at the low-temperature limit (*T* = 0.1 K) is fitted by a power-law singular function at *x* = *x*_*c*_ ≈ 0.03, *C*/*T*∣_*T*=0.1 K_ ~ ∣*x* − *x*_*c*_∣^−*β*^, with *β* ≈ 0.11 for *x* < *x*_*c*_ and *β* ≈ 0.16 for *x* > *x*_*c*_ (pink curves, right axis). Data of **c** is measured at *B* = 90 kOe. The error bar of *C*/*T* comes from the uncertainty of tuning temperature.

Single crystals Ce_1−*x*_Nd_*x*_CoIn_5_ were grown and characterized in ref. ^26^. Previous studies showed that antiferromagnetism coexists with superconductivity for 0.02 < *x* < 0.17^26^. Here, we investigate further the Planckian state by applying a magnetic field in this material close to the edge of antiferromagnetism at *x* ≈ 0.02. The electrical resistivity and heat capacity measurements were performed in a quantum design PPMS-9 system. The doping-field-temperature (*x*, *B*, *T*) phase diagram of this material is shown in Fig. 1a. Doping Nd into the pure CeCoIn_5_ effectively introduces chemical pressure and reduces Kondo coupling, therefore favors the long-range antiferromagnetism^25,26^. At zero magnetic field, pure *d*-wave superconducting ground state exists for very low Nd doping 0 < *x* ≤ 0.02^27^, followed by a co-existing antiferromagnetic superconducting state for 0.05 ≤ *x* < 0.17, and the long-ranged antiferromagnetic phase is reached for *x* > 0.17^26^. For 0 < *x* < 0.17, the resistivity at finite temperatures shows a maximum at temperature *T*_coh_ ~ 45K where coherent Kondo hybridization is reached, a generic feature of many heavy-fermion superconductors, it then drops to zero at 1K < *T*_*c*_ < 2K where superconductivity emerges^20,26^.

**Fig. 2:**
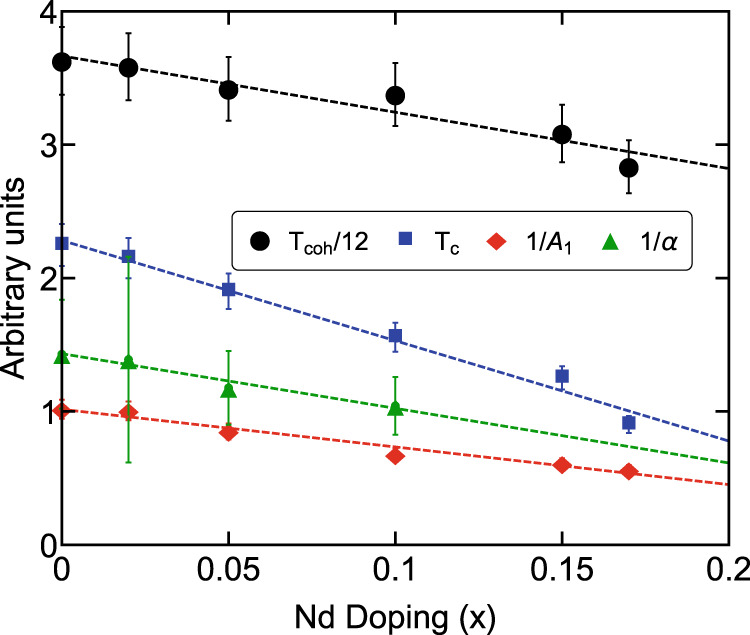
Linear-in-*x* dependence of *T*_coh_, *T*_*c*_ and 1/*A*_1_, and 1/*α*. The data are taken from ref. ^26^ and Table 1. The error bars of *T*_coh_, *T*_*c*_ and 1/*A*_1_ arise from the inaccuracy of tuning temperature, while the error bar for 1/*α* includes the uncertainties of *m*^⋆^, *n*, and *F* shown in Table 1.

The SM behavior with linear-in-*T* resistivity was observed in the intermediate temperature range *T*_*c*_ < *T* < 20K: *ρ*(*T*) = *ρ*_0_ + *A*_1_*T* with *ρ*_0_ being residual resistivity extrapolated to zero temperature and *A*_1_(*α*) being the slope. Via quantum oscillation experiments in ref. ^10^, the scattering rate 1/*τ* for *x* = 0 extracted via the Drude formula, *ρ* = *m*^⋆^/*ne*^2^*τ*, from the resistivity data combined with the carrier concentration *n*_*i*_, electron effective mass ${m}_{i}^{\star }$ associated with the band *i* = *α*,  *β*-band in the *T*-linear resistivity region has been shown to reach the Planckian dissipation limit, $\alpha=\hbar/{k}_{B}T\tau=({e}^{2}\rho /{k}_{B}T){\sum }_{i}{n}_{i}/{m}_{i}^{\star }={A}_{1}({e}^{2}/{k}_{B}){\sum }_{i}({n}_{i}/{m}_{i}^{\star }) \sim 1$. In Table 1, we find similar values of *α* ~ *O*(1) for *x* = 0, 0.02, 0.05 by a distinct set of quantum oscillation measurements in ref. ^28^ (detailed analysis in Supplementary Notes 2 and 3). Interestingly, as shown in Fig. 2, we find the inverse slope 1/*A*_1_, *T*_*c*_ and *T*_coh_ all show linear dependence on *x* over 0 < *x* < 0.15. In particular, *T*_coh_, depending mostly on the Kondo scale, is well approximated by a perfect linear relation to the inverse slope 1/*A*_1_ as well as 1/*α*,

1 $${T}_{{{{{{{{\rm{coh}}}}}}}}}(x)\propto 1/{A}_{1}(x)\propto 1/\alpha (x),$$

up to a non-universal constant shift. Eq. (1) above suggests that the Planckian coefficient *α* inversely depends on the strength of Kondo hybridization in the Planckian SM region, as the second possible scenario mentioned above. To further investigate how *α* depends on Kondo hybridization near the critical doping *x*_*c*_ and its relation to the possible QCP hidden inside the superconducting dome, we apply an external magnetic field and study signatures of quantum critical scaling in resistivity and specific heat coefficient.

**Table 1:** The band-specific parameters of Ce_1−*x*_Nd_*x*_CoIn_5_

	*x* = 0^∘^ (*θ* = 0)	*x* = 0.02 (*θ* = 3^∘^)	*x* = 0.05 (*θ* = 2^∘^)	*x* = 0.1 (*θ* = 7^∘^)
*F*(kT) (*α* band)	4.9 ± 0.5	4.89 ± 1.02	4.88 ± 0.5	4.41 ± 0.41
*n*( × 10^28^m^−3^) (*α* band)	0.31 ± 0.04	0.32 ± 0.07	0.26 ± 0.02	0.17 ± 0.02
*m*^⋆^(*m*_0_) (*α* band)	11.7 ± 2.6	11.7 ± 4.3	9.15 ± 2.0	7 ± 1.0
*A*_1_(*μ*Ω ⋅ cm/K)	0.98 ± 0.07	1.0 ± 0.07	1.17 ± 0.08	1.49 ± 0.1
*α*(*α* band + *β* band)	0.7 ± 0.2	0.72 ± 0.4	0.85 ± 0.2	0.96 ± 0.2

The averaged dHvA frequencies *F*, carrier concentration *n*, and effective masses *m*^⋆^ of the *α* band shown here are taken from ref. ^28^. Carrier concentration *n* of the *α*-band for *x* = 0.05 is estimated by a *d*-dimensional Fermi volume with *d* = 2.45, while it is estimated by a two-dimensional Fermi volume for *x* = 0,  0.02, in accordance with the angular dependence of the dHvA frequencies measured in ref. ^26^. Here, *m*_0_ denotes the bare electron mass, while *α* and *A*_1_ are extracted from the linear-in-temperature resistivity at zero field from ref. ^26^. The error bars of *F*, *n*, and *m*^⋆^ represent the standard deviation. The error bars of *α* and *A*_1_ shown here are the same as described in Fig. 2. Note that the *α* coefficient shown here contains a sub-leading contribution from the *β* band, estimated by the band parameters for pure CeCoIn_5_ in Ref. ^10^: *n* ≈ 0.63 × 10^28^m^−3^ (corresponding to the average dHvA frequency *F* ≈ 9.75 kT) and *m*^⋆^ ≈ 100*m*_0_ (Supplementary Notes 2 and 3 for details).

**Fig. 3:**
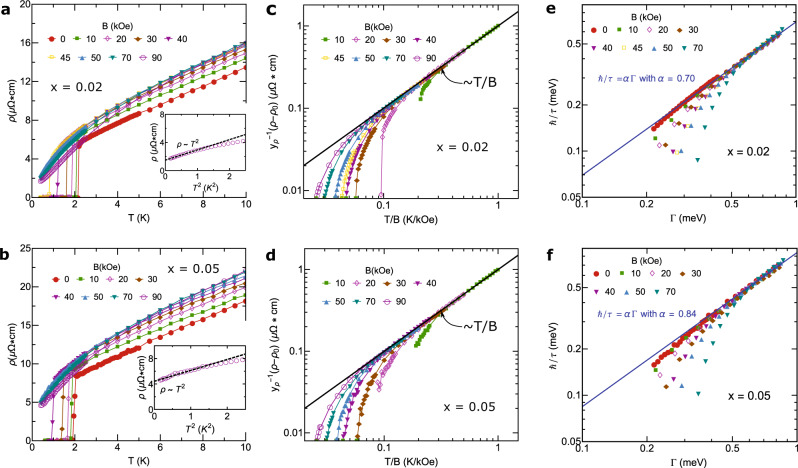
Electrical resistivity and the scaling. **a**, **b** The resistivity *ρ*(*T*) of Ce_1−*x*_Nd_*x*_CoIn_5_ with *x* = 0.02 and *x* = 0.05, respectively, for *B*∥*c*. The insets of **a**, **b** show the Fermi-liquid behavior with *ρ*(*T*) ~ *T*^2^ electrical resistivity at low temperatures for *B* = 90 kOe. **c**, **d** The *T*/*B* scaling of both **a** and **b** show an universal function linearly proportional to *T*/*B* (black solid lines). *y*_*ρ*_ is a non-universal (field-dependent) constant, satisfying ${y}_{\rho }^{-1}[\rho (T)-{\rho }_{0}]=T/B$. **e**, **f** The scattering rates ℏ/*τ* for *x* = 0.02,   0.05 at the intermediate temperature regime where the electrical resistivity exhibits a linear-in-*T* dependence show an universal scaling function ℏ/*τ* = *α*Γ (blue solid lines), with $\Gamma \equiv \sqrt{{({k}_{B}T)}^{2}+{(l{\mu }_{B}B)}^{2}}$, *τ* being the relaxation time, *μ*_*B*_ being the Bohr magneton, and *l* = 0.67 being a fitting parameter^43^. In **e** and **f**, the *β* band of pure CeCoIn_5_, with the same band parameters used in Table 1, is taken into account in performing the Γ scaling of resistivity. The extracted *α* coefficients shown in **e** and **f** are consistent with that shown in Table 1.

**Fig. 4:**
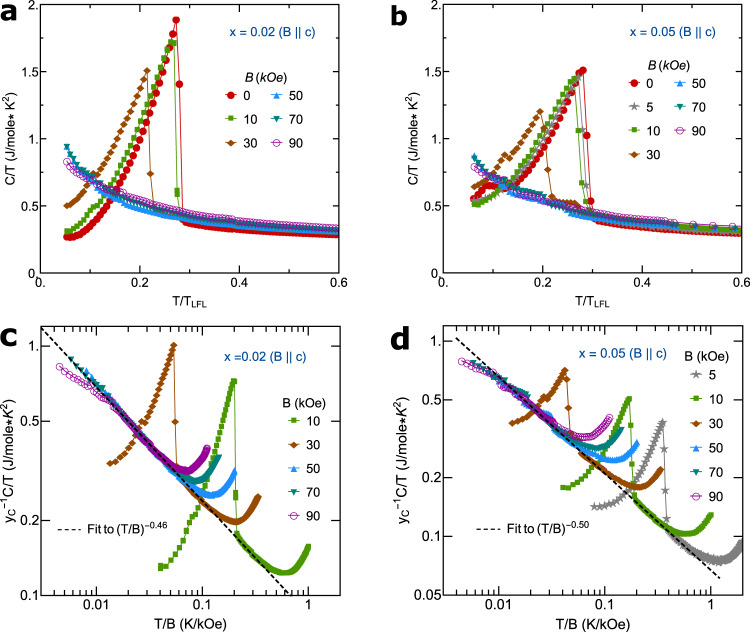
Specific heat coefficient *C*/*T* and its *T*/*B* scaling. **a**, **b** show the electronic specific heat coefficient *C*/*T* versus *T*/*T*_*LFL*_ with different fields *B*∥*c* for 0.02, and 0.05, respectively while **c** and **d** display the power-law *T*/*B* scaling of **a** and **b**. Here, *y*_*C*_ in **c** and **d** represents a non-universal scaling parameter, obeying ${y}_{C}^{-1}(C/T)=\Phi (T/B)$ with Φ being a power-law function here.

As shown in Fig. 3a, b, for *x* = 0.02 and *x* = 0.05, the linear-in-*T* resistivity shows almost the same slopes (*A*_1_) for 0 ≤ *B* ≤ 70 kOe. This strongly indicates that the Kondo hybridization strength approximately remains at a constant quantum critical fixed point value, associated with a QCP for 0.02 < *x*_*c*_ < 0.05 at zero field or a quantum critical line for the above field range (Fig. 1a). The scenario of the QCP associated with the critical Kondo hybridization is further supported by the following evidences: (i) quantum-critical *T*/*B* scaling behavior in the linear-in-*T* resistivity regime (Fig. 3c, d), (ii) the universal Planckian scaling of electron scattering rate ℏ/*τ* = *α*Γ with $\Gamma=\sqrt{{({k}_{B}T)}^{2}+{(l{\mu }_{B}B)}^{2}}$ with *α* ~ *O*(1) (Fig. 3e, f), (iii) the *T*/*T*_*LFL*_-scaling in specific heat coefficient *γ*(*T*/*T*_*LFL*_) ≡ *C*/*T* (Fig. 4a, b) with *T*_*LFL*_ being the Fermi-liquid crossover scale, (iv) the *T*/*B*-power-law scaling in the normal state *γ*-coefficient: *γ* ~ Φ(*T*/*B*) where Φ(*z*) ~ *z*^−*m*^ is an universal scaling function with *m* ≈ 0.46 for *x* = 0.02 (Fig. 4a, c) and *m* ≈ 0.5 for *x* = 0.05 (Fig. 4b, d), and (v) power-law singular specific heat coefficient in the SM state: $C/T{\left|\right.}_{T=0.1{{{{{{{\rm{K}}}}}}}}} \sim|x-{x}_{c}{|}^{-\beta }$ with *β* ≈ 0.11 for *x* < *x*_*c*_ and *β* ≈ 0.16 for *x* > *x*_*c*_ (Fig. 1c). A more accurate estimation of the location of this hidden QCP reveals *x* = *x*_*c*_ ≈ 0.03 by extrapolating the singular specific heat coefficient in the SM state under fields as Nd doping is tuned across the transition (Fig. 1c)^4,29^. When superconductivity is fully suppressed by magnetic field, a crossover from the non-Fermi liquid SM to the Fermi-liquid state with *T*^2^ resistivity is clearly observed on two sides of the transition (*x* = 0.02 and *x* = 0.05) (Fig. 3a, b). This indicates a putative quantum critical (QC) line extended from the QCP hidden under superconducting dome at zero field and Nd doping (Fig. 1a)^25^.

To qualitatively understand the above strange metal behaviors and more importantly the relation between Planckian coefficient *α* and Kondo hybridization near the QCP at *x*_*c*_, here we propose a microscopic mechanism. It is based on the interplay between Kondo screening of a local *f*-electron (*f*_*iσ*_ on site *i* with spin *σ*), by mobile electrons (*c*_*iσ*_), and the AF correlations between nearest-neighbor spins of local *f*-electrons in the form of quasi-2*d* (*d* = 2 + *η* with 0 < *η* < 1) Kondo-Heisenberg (KH) model (see “Methods” section). This mechanism has been used to qualitatively describe the AF QCP at *x*_*c*_ inside the superconducting dome of CeRhIn_5_ under pressure^30,31^, which bears a striking similarity to that for Ce_1−*x*_Nd_*x*_CoIn_5_ at zero field under Nd doping. The Anderson’s resonating-valence-bond (RVB) spin-liquid state^32^, defined by the spatially homogeneous bosonic spin-singlet pair, ${\Delta }_{RVB}\equiv {J}_{H}{\sum }_{\sigma }\langle {{{{{{{\rm{sgn}}}}}}}}(\sigma ){f}_{i\sigma }^{{{{\dagger}}} }{f}_{j,-\sigma }^{{{{\dagger}}} }\rangle$, on nearest-neighbor sites *i*,  *j*, is introduced here for the AF Heisenberg term with the exchange coupling *J*_*H*_. The Kondo hybridization is described by the spatially homogeneous bosonic *χ* field where, at the mean-field level, $\chi \equiv {J}_{K}{\sum }_{\sigma }\langle {f}_{i\sigma }^{{{{\dagger}}} }{c}_{i\sigma }\rangle$ with Kondo coupling *J*_*K*_. At the edge of antiferromagnetism, the Kondo effect not only stablizes the RVB spin liquid against the magnetic long-ranged order by partially sharing the *f*-electron spins^33^, but also introduces hoping of the RVB bonds to the conduction band to form charged Cooper pairs. This leads to a Kondo-RVB coexisting heavy-electron superconducting state with estimated transition temperature *T*_*c*_ ~ *χ*^2^Δ_*RVB*_, in quantitatively good agreement with the experimental observation^34^.

Via the competition between Kondo and RVB physics, an AF-KB QCP was predicted inside the superconducting dome, qualitatively describing the phase transition between AF-superconducting coexisting phase and a pure superconducting phase observed in CeRhIn_5_^34^, similar to our case here. By analyzing the amplitude fluctuations of the Kondo and RVB correlations beyond mean-field level via renormalization group (RG) analysis^34,35^, this mechanism provides a qualitative and semi-quantitative understanding of the SM properties in CeCoIn_5_ in fields and pressure near the AF-KB QCP^20,24,25^. In particular, it captures the observed *T*-linear resistivity in terms of critical Kondo (charge) fluctuations ($\hat{\chi }$ field) via the electron-phonon-like interaction, ${\hat{H}}_{K} \sim {J}_{K}{\sum }_{i\sigma }({c}_{i\sigma }^{{{{\dagger}}} }{f}_{i\sigma }{\hat{\chi }}_{i}+H.c.)$ (see “Methods” section), and a power-law-in-*T* divergence in *γ*(*T*) via both Kondo and RVB fluctuations^34,35^. The quasi-2*d* nature of our theoretical framework, essential to the KB transition, is consistent with the quasi-2*d* nature of the Fermi surface in CeCoIn_5_^36^ as well as the dimensional crossover in Fermi surface evolution of the *α*-band from 2*d*-like to 3*d*-like in our system^28^ with increasing Nd concentrations observed in quantum oscillations. By analyzing the data of quantum oscillation in ref. ^28^, we observed a sudden change in charge carrier *n* across *x*_*c*_ as *T* → 0 (Table 1), a possible signature of a jump in Fermi surface volume at ground state. This signature is consistent with our KB QCP scenario. Interestingly, evidence of a delocalization (KB) transition has been observed in a closely related compound CeCo(In_1−*x*_Sn_*x*_)_5_ near *x* ~ 0.016^37^. It is promising to expect that the KB QCP might also occur here.

We now apply the above theoretical framework to investigate how the Planckian coefficient *α* depends on the Kondo hybridization. First, the coherent Kondo temperature *T*_coh_ is related to the condensation amplitude of Kondo hybridization *χ* through *T*_coh_ ~ *χ*^2^/*D*^34,38^. Since *χ* is proportional to the Kondo coupling *J*_*K*_, this suggests ${T}_{{{{{{{{\rm{coh}}}}}}}}} \sim {J}_{K}^{2}/D$. From Eq. (1), we have

2 $$\alpha (x)\propto {A}_{1}(x)\propto 1/{T}_{{{{{{{{\rm{coh}}}}}}}}}(x)\propto 1/{J}_{K}^{2}$$

up to a non-universal constant shift. Meanwhile, within our theoretical framework, the superconducting transition temperature *T*_*c*_ of an antiferromagnetic quantum critical heavy-fermion superconductor is related to the RVB and Kondo correlations via^34,39^

3 $${T}_{c}\propto {\chi }^{2}{\Delta }_{RVB}\propto {J}_{K}^{2}{\Delta }_{RVB}.$$

The experimentally observed linear relation (up to a non-universal constant) between *T*_*c*_ and the inverse slope 1/*A*_1_: *T*_*c*_(*x*) ∝ 1/*A*_1_(*x*) ∝ 1/*α*(*x*) and Eq. (3) implies that $\alpha \propto 1/{J}_{K}^{2}$, consistent with Eq. (2) above.

The approximated constant value of *α* ~ *O*(1) seen for 0 ≤ *x* ≤ 0.05 (with an error bar, Table 1) can therefore be understood as the Kondo hybridization being close to quantum critical. This is also in good agreement with the same slope observed in linear-in-*T* resistivity near the edge of antiferromagnetism as well as the decreasing 1/*A*_1_ (or Kondo correlation) with increasing Nd doping (Fig. 2). Furthermore, the experimentally and theoretically supported relations between *A*_1_ and the Kondo coupling *J*_*K*_, $\alpha \propto {A}_{1}\propto 1/{J}_{K}^{2}$ strengthen the link between the Planckian *T*-linear scattering rate and critical Kondo hybridization in our system.

A few remarks are made before we conclude. Firstly, in the SM region, the electron effective mass *m*^⋆^ in general may be temperature dependent. In such case, the Planckian scattering rate is reached only near the lower end of the linear-in-*T* resistivity region where *m*^⋆^ is well-approximated by a temperature-independent constant extracted from quantum oscillation measurement in the Fermi-liquid region^17^. Nevertheless, we have checked within our theoretical framework that the temperature dependence of *m*^⋆^ in our SM region is negligible. As a result, the Planckian state in our system extends over the entire SM region. Secondly, though the Drude formula has been widely used to relate the transport and scattering rate in the Fermi-liquid region and near the lower end of the *T*-linear region, whether it is valid deep in the non-Fermi liquid SM region is an open question^17,40^. Thirdly, while quantum oscillation measurement is widely used to estimate *n* and *m*^⋆^, different approach by Hall coefficient and *A* coefficient associated with the Fermi-liquid behavior with *T*^2^ resistivity has been used^40^. Finally, the above quantum critical features in specific heat coefficient are inconsistent with the conventional spin-density-wave (SDW) theory^41,42^ for the AF QCP though the SDW fluctuations are expected to appear.

In summary, we observe the quantum-critical *T*/*B*-scaling of the Planckian metal state in the resistivity and specific heat coefficient of heavy-electron superconductor Ce_1−*x*_Nd_*x*_CoIn_5_ under fields. Clear experimental evidences are shown to support the notion of Kondo hybridization being quantum critical at *x*_*c*_ ~ 0.03. We propose a microscopic mechanism based on the quasi-2*d* Kondo-Heisenberg lattice model to qualitatively account for the observed strange metal behaviors. Furthermore, we find that the Planckian coefficient *α* for Nd-doped CeCoIn_5_ shows an inversely quadratic dependence in the Kondo coupling, as inferred from our theoretical framework as well as the linear relation between 1/*α*, the inverse slope of *T*-linear resistivity 1/*A*_1_ and the coherence temperature *T*_coh_. Our observation and proposed mechanism offer the first microscopic understanding of the Planckian dissipation limit in a quantum critical system. This generic mechanism is relevant for other heavy-fermion quantum critical superconductors showing Planckian metal states, such as CeRhIn_5_ and related compounds. Our results motivate further experimental study, such as the Hall coefficient measurement on the signatures of Kondo breakdown near the critical Nd doping as well as theoretical studies, such as more accurate predictions on the value of *α* and crit ical exponents in scaling behaviors of observables, and a fundamental issue on whether quantum critical systems with *T*-linear resistivity, in general, implies the Planckain metals.

## Methods

### A microscopic model

Our start point is the microscopic large-*N* (Sp(*N*)) Kondo-Heisenberg Hamiltonian *H* = *H*_0_ + *H*_*K*_ + *H*_*J*_, where

4 $${H}_{0}=	 \mathop{\sum}\limits_{\langle i,j\rangle ;\sigma }\left[{t}_{ij}{c}_{i\sigma }^{{{{\dagger}}} }{c}_{j\sigma }+H.c.\right]-\mathop{\sum}\limits_{i\sigma }\mu \,{c}_{i\sigma }^{{{{\dagger}}} }{c}_{i\sigma },\\ {H}_{J}=	 \mathop{\sum}\limits_{\langle i,j\rangle }\frac{{J}_{ij}}{N}{{{{{{{{\bf{S}}}}}}}}}_{i}^{f}\cdot {{{{{{{{\bf{S}}}}}}}}}_{j}^{f},\quad {H}_{K}=\frac{{J}_{K}}{N}\mathop{\sum}\limits_{i}{{{{{{{{\bf{S}}}}}}}}}_{i}^{f}\cdot {{{{{{{{\bf{s}}}}}}}}}^{c}.$$

In Eq. (4), *H*_0_ describes hopping of conduction (*c*_*iσ*_) electrons. The antiferromagnetic Heisenberg interaction between two impurity-spin (**S**^*f*^) of the electrons occupying the localized *f* orbitals of neighboring sites is described by *H*_*J*_ and the Kondo screening of impurity spins by conduction electrons is captured by *H*_*K*_. Via Hubbard-Stratonovich transformation, *H*_*K*_ and *H*_*J*_ can be factorized as

5 $${H}_{J}\to 	 \mathop{\sum}\limits_{\langle i,j\rangle ;\alpha,\beta }\left[{\Phi }_{ij}{{{{{{{{\mathscr{J}}}}}}}}}^{\alpha \beta }{f}_{i\alpha }{f}_{j\beta }+H.c.\right]+\mathop{\sum}\limits_{\langle i,j\rangle }N\frac{|{\Phi }_{ij}{|}^{2}}{{J}_{H}},\\ {H}_{K}\to 	 \frac{1}{\sqrt{N}}\mathop{\sum}\limits_{i,\sigma }\left[\left({c}_{i\sigma }^{{{{\dagger}}} }{f}_{i\sigma }\right){\chi }_{i}+H.c.\right]+\mathop{\sum}\limits_{i}\frac{|{\chi }_{i}{|}^{2}}{{J}_{K}}.$$

Here, the local spin operator ${{{{{{{{\bf{S}}}}}}}}}_{i}^{f}$ is represented in terms of the constraint fermionic Sp(*N*) fields, *f*_*iα*_, which needs to be subjected to the constraint, $\langle {\sum }_{\sigma }{f}_{i\sigma }^{{{{\dagger}}} }{f}_{i\sigma }\rangle=N\kappa$, to ensure its local nature. The constant *κ* < 1 allows us to capture the valence fluctuations. Thus, to capture the local constraint, an additional term has to be included in the Hamiltonian, ${H}_{\lambda }={\sum }_{i,\sigma }\lambda \left[{f}_{i\sigma }^{{{{\dagger}}} }{f}_{i\sigma }-Q\right]$ with *λ* being the Lagrange multiplier. In the *H*_*J*_ term, we assume an uniform antiferromagnetic RKKY coupling *J*_*ij*_ = *J*_*H*_ on a lattice where *i*, *j* are nearest-neighbor site indices, ${{{{{{{{\mathscr{J}}}}}}}}}^{\alpha \beta }={{{{{{{{\mathscr{J}}}}}}}}}_{\alpha \beta }=- \!\! {{{{{{{{\mathscr{J}}}}}}}}}^{\beta \alpha }$ denotes the anti-symmetric tensor, $\sigma,\alpha,\beta \in \{-\frac{N}{2},\cdots \,,\frac{N}{2}\}$ label the spin indices. Here, *χ*_*i*_ and Φ_*ij*_ are the spatially dependent Hubbard-Stratonovich fields, where their (spatially uniform) mean-field values, $\chi \equiv \langle \frac{{J}_{K}}{\sqrt{N}}{\sum }_{\sigma }{f}_{i\sigma }^{{{{\dagger}}} }{c}_{i\sigma }\rangle$ and ${\Delta }_{RVB}\equiv \langle {\Phi }_{ij}\rangle=\langle \frac{{J}_{H}}{N}{\sum }_{\alpha,\beta }\,{{{{{{{{\mathscr{J}}}}}}}}}_{\alpha \beta }{f}_{i}^{\alpha {{{\dagger}}} }{f}_{j}^{\beta {{{\dagger}}} }\rangle$, can be used as order parameters to describe the Kondo-screened heavy Fermi liquid state and the RVB spin-liquid state.

While considering the fluctuations of *χ*_*i*_ and Φ_*ij*_ beyond mean-field level, we can express ${\chi }_{i}\to \chi+{J}_{K}{\hat{\chi }}_{i}$ and ${\Phi }_{ij}\to {\Delta }_{RVB}+{J}_{H}{\hat{\Phi }}_{ij}$, where ${\hat{\chi }}_{i}$ and ${\hat{\Phi }}_{ij}$ denote the fluctuation of *χ* and Δ_*RVB*_. Both *H*_*K*_ and *H*_*J*_ can be divided into the mean-field and fluctuating parts, namely ${H}_{K}\to {H}_{K}^{MF}+{\hat{H}}_{K}$ and ${H}_{J}\to {H}_{J}^{MF}+{\hat{H}}_{J}$ with ${H}_{J}^{MF}$ and ${H}_{K}^{MF}$ at mean-field, and ${\hat{H}}_{J}$ and ${\hat{H}}_{K}$ beyond mean-field level. The microscopic model relevant for the heavy-fermion materials therefore becomes $H\to {H}_{0}+{H}_{\lambda }+{H}_{J}^{MF}+{H}_{K}^{MF}+{\hat{H}}_{K}+{\hat{H}}_{J}$.

## Supplementary information

Supplementary Information

Peer Review File

## Data Availability

Original data and codes created for the study have been deposited in the online Zenodo repository (10.5281/zenodo.7187946).
