# Supplementary material for: The scaled-invariant Planckian metal and quantum criticality in Ce1−xNdxCoIn5
Source: Nat Commun. 2023 Feb 3;14:581. doi: 10.1038/s41467-023-36194-9 (PMC9898561; doi:10.1038/s41467-023-36194-9)
Supplement: Supplementary file 1 — Supplementary Information [file 41467_2023_36194_MOESM1_ESM.pdf]

Supplementary Information of "The scaled-invariant Planckian metal and quantum criticality in $Ce_{1-x}Nd_xCoIn_5$"

Yung-Yeh Chang[1,2], Hechang Lei[3,4], C. Petrovic[3,†], and Chung-Hou Chung[1,2,‡]

[1]Physics Division, National Center for Theoretical Sciences, Taipei 10617, Taiwan, Republic of China
[2]Department of Electrophysics, National Yang-Ming Chiao-Tung University, Hsinchu, 300 Taiwan, R.O.C.
[3]Condensed Matter Physics and Materials Science Department, Brookhaven National Laboratory, Upton, New York 11973-5000, USA
[4]Present Address: Department of Physics and Beijing Key Laboratory of Opto-electronic Functional Materials & Micro-nano Devices, Renmin University of China, Beijing, China
[†]Corresponding author: petrovic@bnl.gov
[‡]Corresponding author: chung0523@nycu.edu.tw

ABSTRACT

In this Supplementary Information, we provide additional details and derivations that are not included in the main text.

Supplementary Note 1: Specific heat coefficient and its scaling for zero Nd doping ($x = 0$)

Supplementary Figure1 shows the specific heat coefficient and its scaling at zero Nd doping ($x = 0$) under different magnetic fields. A T/B-power-law scaling behavior within the quantum-critical regime, $\gamma(T/B) \sim (T/B)^{-m}$, with exponent $m = 0.51$, closed to the exponents for $x = 0.02$ and $x = 0.05$, is also found here (Supplementary Figure1b).

Supplementary Note 2: Estimation of carrier concentration n and α coefficient

In this section, we provide derivation of the relevant equations for carrier concentration n based on the quantum oscillation measurements. We will further use those equations to reproduce n and the Planckian coefficients α shown in Table 1 of the main text.

We start from the formula of quantum oscillation frequency F, given by

$$F = \frac{(h/2\pi)}{2\pi e} S_F \tag{S1}$$

with S_F being the extremal cross-sectional area of the Fermi surface. For simplicity, we assume a circular cross-section of Fermi surface here, hence $S_F = \pi k_F^2$ with k_F being the "averaged" Fermi wave vector of the circular Fermi surface. This links the dHvA frequency and the "average" Fermi wave vector of the Fermi surface by $F = \frac{(h/2\pi)k_F^2}{2e}$. Here, we can make a link of F and the carrier concentration n through k_F. While considering the effective dimensionality of critical modes, the carrier concentration takes the following form[1]:

$$\begin{aligned} n &= \frac{2k_F}{\pi d_b d_c} = \frac{2}{\pi d_b d_c}\sqrt{\frac{2eF}{h/2\pi}} \quad \text{(for 1d)}, \\ n &= \frac{k_F^2}{2\pi d_c} = \frac{1}{2\pi d_c}\left(\frac{2eF}{h/2\pi}\right) \quad \text{(for 2d)}, \\ n &= \frac{k_F^3}{3\pi^2} = \frac{1}{3\pi^2}\left(\frac{2eF}{h/2\pi}\right)^{3/2} \quad \text{(for 3d)}, \end{aligned} \tag{S2}$$

where d_b and d_c are the lattice constants of unit cell along the b and c axes. Note that, for the $2d$ case of Eq. (S2), we assume the system has a strong anisotropy along the c direction while it remains isotropic in the a-b plane.

As an example, we provide detailed derivation of carrier concentration for the $2d$ case shown in Eq. (S2) and then generalize this derivation to the case with fractional quasi-$2d$ dimension.

Assume the critical modes occur on the isotropic ab-plane. The total number of states can be expressed as

$$\begin{aligned} N &= N_{ab}N_c = 2\times\frac{\pi k_F^2}{\Delta V_k}\times\frac{L_c}{d_c} = 2\times\frac{A\pi k_F^2}{4\pi^2}\frac{L_c}{d_c} \\ &\rightarrow n = \frac{N}{\mathscr{V}} = \frac{N}{AL_c} = \frac{k_F^2}{2\pi d_c}, \end{aligned} \tag{S3}$$

where $N_{ab} = \frac{\pi k_F^2}{\Delta V_k}$ is the number of states on the ab plane per spin while $N_c = L_c/d_c$ for that along the c-axis. Here, ΔV_k represents the unit volume in k space occupied by a state, L_c denotes the sample site along c, $A = L^2$ is the area of the sample on the ab plane. This indicates the total volume of the sample $\mathscr{V} = AL_c$.

The above approach of deriving the carrier concentration for the effective $2d$ critical modes embedded in a $3d$ lattice can

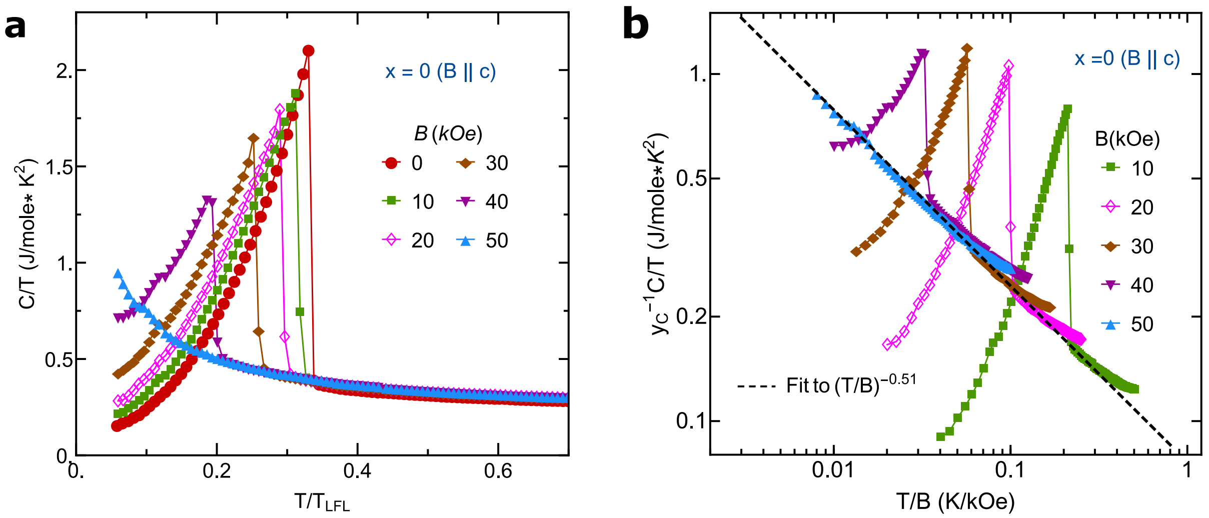

Supplementary Figure 1. Specific heat coefficient C/T and its scaling for zero Nd doping ($x=0$). a shows the electronic specific heat coefficient C/T with different fields $B \parallel c$ for zero Nd doping ($x=0$) while **b** displays the power-law T/B scaling of **a**.

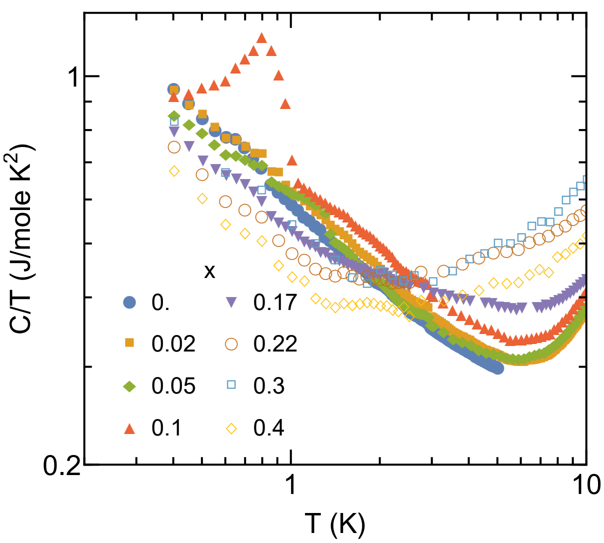

Supplementary Figure 2. Specific heat coefficient C/T at a finite field. Specific heat coefficient at a finite field ($B = 70$kOe along the c axis) with different Nd concentrations (x). Note that C/T for $x=0$ is measured under $B = 50$ kOe.

be generalized for the case of arbitrary dimensional critical modes with fractional quasi-2d dimensionality embedded in a 3d lattice.

The total number of states per spin for an isotropic d-dimensional system is given by

$$N = \sum_{|\mathbf{k}|\leq k_F} \Theta(\varepsilon_{\mathbf{k}}) = \frac{\int_0^{k_F} d^d k}{\Delta V_k} = \frac{V_d(k_F)}{\Delta V_k}, \tag{S4}$$

where $\Delta V_k = (2\pi/L)^d = (2\pi)^d/V_d$ with $V_d \equiv L^d$ and $V_d(k_F)$ is the volume of a d-dimensional sphere with radius k_F,

$$V_d(k_F) = \frac{\pi^{d/2}}{\Gamma(\frac{d}{2}+1)} k_F^d. \tag{S5}$$

In the above equation, $\Gamma(x)$ denotes the Γ function.

For a general d-dimensional critical modes embedded in a 3d lattice, the total number of states reads

$$N = 2 \times N_{ab}^{(d)} \times N_c^{(3-d)}, \tag{S6}$$

where the prefactor 2 comes from the spin degrees of freedom. Here, we assume that the quasi-2d critical modes mostly arise from the ab-plane.

From Eq. (S4), we have

$$N_{ab}^{(d)} = \frac{\frac{\pi^{d/2}}{\Gamma(\frac{d}{2}+1)} k_F^d}{\frac{(2\pi)^d}{V_d}} = \frac{\pi^{d/2} V_d k_F^d}{(2\pi)^d \times \Gamma(\frac{d}{2}+1)}, \tag{S7}$$

while

$$N_c^{(3-d)} = \left(\frac{L_c}{d_c}\right)^{3-d} \tag{S8}$$

The total number of states is then given by

$$N = \frac{\mathscr{V} k_F^d}{2^{d-1}\pi^{d/2} d_c^{3-d}\Gamma(\frac{d}{2}+1)}, \tag{S9}$$

giving rise to the carrier concentration

$$n = \frac{N}{\mathscr{V}} = \frac{k_F^d}{2^{d-1}\pi^{d/2} d_c^{3-d}\Gamma(\frac{d}{2}+1)}. \tag{S10}$$

Using the relation of k_F and F, we obtain the expression of carrier concentration for arbitrary d-dimensional critical modes,

$$n = \frac{1}{2^{d-1}\pi^{d/2}d_c^{3-d}\Gamma(\frac{d}{2}+1)}\left(\frac{2eF}{h/2\pi}\right)^{\frac{d}{2}}. \qquad \text{(S11)}$$

When taking $d=2$, the above expression of n goes back the $2d$ case in Eq. (S2).

Supplementary Note 3: Estimating the Planckian coefficients α for $Ce_{1-x}Nd_xCoIn_5$

Below, we estimate the carrier concentration n and Planckian coefficients α shown in Table 1 of the main text for $Ce_{1-x}Nd_xCoIn_5$ with $x = 0, 0.02, 0.05$, and 0.1 using the dHvA frequency F and effective mass $m^\star$ in Ref.[2] (for α-band) and in Refs.[1,3] (for β-band).

- **For $x=0$.** The average dHvA frequency for the α-band is $F = 4.9$kT while $m^\star = 11.7m_0$ is its average effective mass. Since Fermi surface of pure $CeCoIn_5$ has been shown to be $2d$-like, we thus use the equation of the $2d$ version in Eq. (S2) to estimate the carrier density, given by

$$\begin{aligned} n =& \frac{(2\times1.6\times10^{-19})\times(4.9\times10^{3})}{(7.549\times10^{-10})\times(6.6\times10^{-34})}\text{m}^{-3} \\ =& 0.31\times10^{28}\text{m}^{-3} \quad (\text{for } \alpha\text{-band}). \end{aligned} \qquad \text{(S12)}$$

  For the β-band of pure $CeCoIn_5$, we adopt its Fermi surface parameters: $F = 9.75$kT and $m^\star = 100m_0$. Following the similar approach, the carrier concentration for β-band is

$$n = 0.63\times10^{28}\text{m}^{-3} \quad (\text{for } \beta\text{-band}). \qquad \text{(S13)}$$

  The Planckian coefficient from the α-band can be straightforwardly obtained via $\alpha = \frac{e^2(h/2\pi)}{k_B}A_1\frac{n}{m^\star}$, suggesting

$$\begin{aligned} \alpha =& \frac{(1.6\times10^{-19})^2\cdot(0.98\times10^{-8})\,(0.31\times10^{28})}{(1.38\times10^{-23})\cdot(11.7\times9.11\times10^{-31})} \\ & \times(1.05\times10^{-34}) \\ =& 0.56 \quad (\text{for } \alpha\text{-band}), \end{aligned} \qquad \text{(S14)}$$

  and, for the β-band, we have

$$\alpha = 0.13 \quad (\text{for } \beta\text{-band}). \qquad \text{(S15)}$$

  These two contributions give $\alpha \approx 0.7$ for pure $CeCoIn_5$. The A_1 coefficient for pure $CeCoIn_5$ at zero field, $A_1 = 0.98\mu\Omega\cdot$cm/K, is used for the estimation of the α coefficients shown above.

- **For $x=0.02$.** The average dHvA frequency for the α-band is $F = 4.89$kT while $m^\star = 11.7m_0$ is its average effective mass. Angular dependence of the dHvA frequencies indicates a $2d$ Fermi surface of the α-band for $x = 0.02$[2]. Following the similar approach, the carrier concentration of the α-band is calculated as

$$n = 0.32\times10^{28}\text{m}^{-3} \quad (\text{for } \alpha\text{-band}). \qquad \text{(S16)}$$

  Here, we assume that the carrier density and the relevant band parameters as well as the effective dimension of the β-band do not significantly altered while doping 2% of Nd, indicating that $n = 0.63\times10^{28}\text{m}^{-3}$ and $m^\star = 100m_0$ for the β-band here. The α-coefficients for the α- and β-band are thus estimated

$$\begin{aligned} \alpha &= 0.58 \quad (\text{for } \alpha\text{-band}), \\ \alpha &= 0.14 \quad (\text{for } \beta\text{-band}), \end{aligned} \qquad \text{(S17)}$$

  giving the total Planckian coefficient $\alpha = 0.72$ for $x = 0.02$. The gradient of the linear-T resistivity $A_1 = 1.0\mu\Omega\cdot$cm/K is used in this case.

- **For $x=0.05$.** The fundamental band parameters of the α-band for $x = 0.05$ is $F = 4.88$kT and $m^\star = 9.15m_0$. Angular dependence of the dHvA frequencies indicates a $2d$-to-$3d$ dimensional crossover of Fermi surface of the α-band at $x = 0.05$[2]. Accompanying with the prediction of a QCP at $x_c = 0.03$ and the theoretical studies on that QCP, we treat the dimensionality for $x = 0.05$ to be $d = 2.45$. Using Eq. (S11), the carrier concentration of α-band is estimated as

$$n = 0.26\times10^{28}\text{m}^{-3} \quad (\text{for } \alpha\text{-band}). \qquad \text{(S18)}$$

  Likewise, we assume the band parameters of the β-band also remains the same for $x = 0.05$, thus $m^\star = 100m_0$ and $F = 9.75$kT. The carrier concentration of the β-band with $d = 2.45$ is found to be

$$n = 0.6\times10^{28}\text{m}^{-3} \quad (\text{for } \beta\text{-band}). \qquad \text{(S19)}$$

  Using $A_1 = 1.17\mu\Omega\cdot$cm/K for $x = 0.05$, the α-coefficients for the α- and β-band can be straightforwardly calculated as

$$\begin{aligned} \alpha &= 0.7 \quad (\text{for } \alpha\text{-band}), \\ \alpha &= 0.15 \quad (\text{for } \beta\text{-band}), \end{aligned} \qquad \text{(S20)}$$

  giving the total Planckian coefficient $\alpha = 0.85$.

- **For $x=0.1$.** The fundamental band parameters of the α-band for $x = 0.1$ is $F = 4.41$kT and $m^\star = 7m_0$. We treat the effective dimensionality of Fermi surface of the α-band to be three-dimensional as this compound at $x = 0.1$ is deep inside the AF state[4]. Using Eq. (S11) and

$d = 3$, the carrier concentration of α-band is estimated as

$$n = 0.17 \times 10^{28} \mathrm{m}^{-3} \quad (\text{for } \alpha\text{-band}). \tag{S21}$$

Similarly, we assume that $m^{\star} = 100m_0$ and $F = 9.75$kT are also applicable for the β-band for $x = 0.1$ here. The carrier concentration of the β-band with $d = 3$ is found to be

$$n = 0.55 \times 10^{28} \mathrm{m}^{-3} \quad (\text{for } \beta\text{-band}). \tag{S22}$$

Using $A_1 = 1.49\mu\Omega \cdot \mathrm{cm/K}$ for $x = 0.1$, the α-coefficients for the α- and β-band can be straightforwardly estimated as

$$\begin{aligned} \alpha &= 0.77 \quad (\text{for } \alpha\text{-band}), \\ \alpha &= 0.19 \quad (\text{for } \beta\text{-band}), \end{aligned} \tag{S23}$$

giving the total Planckian coefficient $\alpha = 0.96$.

Supplementary References

1. Bruin, J. A. N., Sakai, H., Perry, R. S. & Mackenzie, A. P. Similarity of Scattering Rates in Metals Showing T-Linear Resistivity. *Science* **339**, 804–807, 10.1126/science.1227612 (2013). https://science.sciencemag.org/content/339/6121/804.full.pdf.

2. Klotz, J. *et al.* Fermi surface reconstruction and dimensional topology change in Nd-doped $CeCoIn_5$. *Phys. Rev. B* **98**, 081105, 10.1103/PhysRevB.98.081105 (2018).

3. Settai, R. *et al.* Quasi-two-dimensional Fermi surfaces and the de Haas-van Alphen oscillation in both the normal and superconducting mixed states of $CeCoIn_5$. *J. Physics: Condens. Matter* **13**, L627–L634, 10.1088/0953-8984/13/27/103 (2001).

4. Hu, R., Lee, Y., Hudis, J., Mitrovic, V. F. & Petrovic, C. Composition and field-tuned magnetism and superconductivity in $Nd_{1-x}Ce_xCoIn_5$. *Phys. Rev. B* **77**, 165129, 10.1103/PhysRevB.77.165129 (2008).
